# Innovating public engagement and patient involvement through strategic collaboration and practice

**DOI:** 10.1186/s40900-019-0160-4

**Published:** 2019-10-21

**Authors:** Leah Holmes, Katharine Cresswell, Susannah Williams, Suzanne Parsons, Annie Keane, Cassie Wilson, Safina Islam, Olivia Joseph, Jahanara Miah, Emily Robinson, Bella Starling

**Affiliations:** Public Programmes Team, Manchester University NHS Foundation Trust, The Nowgen Centre, 29 Grafton Street, Manchester, M13 9WU England

**Keywords:** Patient and public involvement, Public engagement, Health research, Inclusion, Diversity

## Abstract

**Background:**

Patient and public involvement and engagement is an important and expected component of health-related research activity in the UK. Specifically within the health research sphere, public engagement (usually defined as raising awareness of research) and patient involvement (usually defined as actively involving people in research) have traditionally been seen as separate but have much to gain from working together towards a common goal of better health outcomes for all.

**Methods:**

This paper describes a unique approach taken by the Public Programmes Team: a small interdisciplinary team of public engagement specialists, with backgrounds in science, community development, public engagement and involvement, policy, ethics, communications, industry, museums and creative practice, embedded within translational research infrastructure and delivery in Manchester in the North West of England. We propose a new model of professional practice – a 'cycle' of engagement and involvement – innovating across the complementary fields of public engagement and patient involvement, and working inclusively and in partnership with people in health research. Further, our approach capitalises on strategic collaboration offering economies of scale and a joined up way of working. Our ambition is to boldly experiment, learn and reflect, responsibly and based on evidence and partnerships, using methods of engagement that address issues of social justice.

**Results:**

Here, we report on preliminary case studies exemplifying the impact of our approach, and data relating to achievements and learning between April 2017 and March 2018. Informed by our findings, we propose that our approach has the potential to be replicated elsewhere.

**Conclusions:**

Our practice and the beginning of its evaluation lead us to believe that our way of working and model of professional practice – the ‘cycle’ of engagement and involvement – is effective in: addressing our vision of making health research relevant and inclusive for everyone; and embedding and joining up public involvement in a busy and fertile translational health research ecosystem.

**Electronic supplementary material:**

The online version of this article (10.1186/s40900-019-0160-4) contains supplementary material, which is available to authorized users.

## Plain English summary

Working in partnership with people, patients, carers and communities is an important and expected component of health-related research activity in the UK. Within health research, public engagement (usually defined as raising awareness of research) and patient involvement (usually defined as actively involving people in research) have traditionally been seen as separate but have much to gain from working together towards a common goal of better health outcomes for all. This paper describes a unique approach taken by the Public Programmes Team - an interdisciplinary group of public engagement specialists embedded within translational research infrastructure and delivery in Manchester in the North West of England. We propose a new model of professional practice – a 'cycle' of engagement and involvement – innovating across the complementary fields of public engagement and patient involvement, and working inclusively and in partnership with people in health research. Further, our approach capitalises on strategic collaboration offering economies of scale and a joined up way of working. Through preliminary case studies exemplifying the impact of our approach, our achievements and learning between April 2017 and March 2018, we propose that our approach has the potential to be replicated elsewhere. Our ambition is to boldly experiment, learn and reflect, responsibly and based on evidence and partnerships, using methods of engagement that address issues of social justice.

## Introduction

### Background: public engagement versus patient and public involvement?

“A diverse and inclusive public involvement community is essential if research is relevant to population needs and provides better health outcomes for all.” Going the Extra Mile 2015 [1].

Patient and public involvement and engagement is an important and expected component of health-related research activity in the UK. The definitions of public engagement (PE) and patient public involvement (PPI) in the UK (Box 1) have traditionally led to PE being viewed as awareness raising, sharing, informal learning, debate and dialogue activities; and PPI as more formalised partnerships and processes to influence health research.

**Box 1:** Definitions of public engagement and patient involvement in the UK

The **National Coordinating Centre for Public Engagement (NCCPE)** [2] definition:	
Public engagement describes the myriad of ways in which the activity and benefits of higher education and research can be shared with the public. Engagement is by definition a two-way process, involving interaction and listening, with the goal of generating mutual benefit.	
**INVOLVE**, the NIHR National Advisory Group on public involvement in NHS, health and social care research definitions:	
Public involvement is research being carried out ‘with’ or ‘by’ members of the public rather than ‘to’, ‘about’ or ‘for’ them. Members of the public are actively involved in research projects and in research organisations.	
Engagement is where information and knowledge about research is provided and disseminated.	

In health research, public engagement and patient involvement in the UK have historically been siloed in, respectively, academic (Higher Education) and health service (National Health Service [NHS]) based research environments, including through distinct funding mechanisms, definitions (Box 1) and communities of practice. For example, the National Institute of Health Research (NIHR) – England’s largest funder of leading-edge health research, focused on the needs of patients and the public – traditionally funds PPI; the Wellcome Trust is the largest funder of PE with science in the UK, and the UK Research Councils routinely fund public engagement within UK Higher Education Institutions. Examples of good practice in PPI and PE abound and the fields have much to gain from working together, not least because they share similar values, challenges and the goal of working towards better health outcomes for all.

Social scientists have long called for scientists and scientific institutions to democratise the production of scientific knowledge (eg. [2]) through engagement, involvement and acknowledging the value of ‘lay’ expertise. Making scientific research more accountable, through involvement and engagement, may address the decline of trust in scientists and scientific institutions, (eg. [3]). Particularly within the arena of translational research, with its potential to yield clinical applications, research is increasingly conceived as multidisciplinary, socially distributed and oriented towards application and use, with an accompanying emphasis on both engagement and involvement [4, 5]. In addition, from a public point of view, arguably, the distinctions between engagement and involvement in health research are artificial.

Recent reports and initiatives highlight the need for:

- Greater innovation and quality across public engagement and patient involvement [1, 6],
- Increased effective collaborations between research organisations and civic society [1, 6] including the importance of approaches to bring together communities of people and practice,
- Enhanced diversity, professional and career development of practitioners across public engagement [6] and patient involvement [1],
- Better inclusion of so-called ‘harder to reach’, ‘underserved’ or ‘seldom-heard’ audiences [1, 7] in health, research and engagement.

We believe that rather than drawing arbitrary distinctions between public engagement and patient involvement, the two disciplines can work together to foster inclusive research communities. We have developed a way of working across PPI and PE that aims to provide complementarity, and innovate the spaces, between PE and PPI, with a view to addressing the above needs. Our ‘cycle’ of PPIE (an umbrella term to describe the wealth of practices in PPI and PE, used in the absence of a better alternative) is based on our expertise and experience across both PPI and PE prior to 2017.

### Background: context of the public Programmes team

**Fig. 1:**
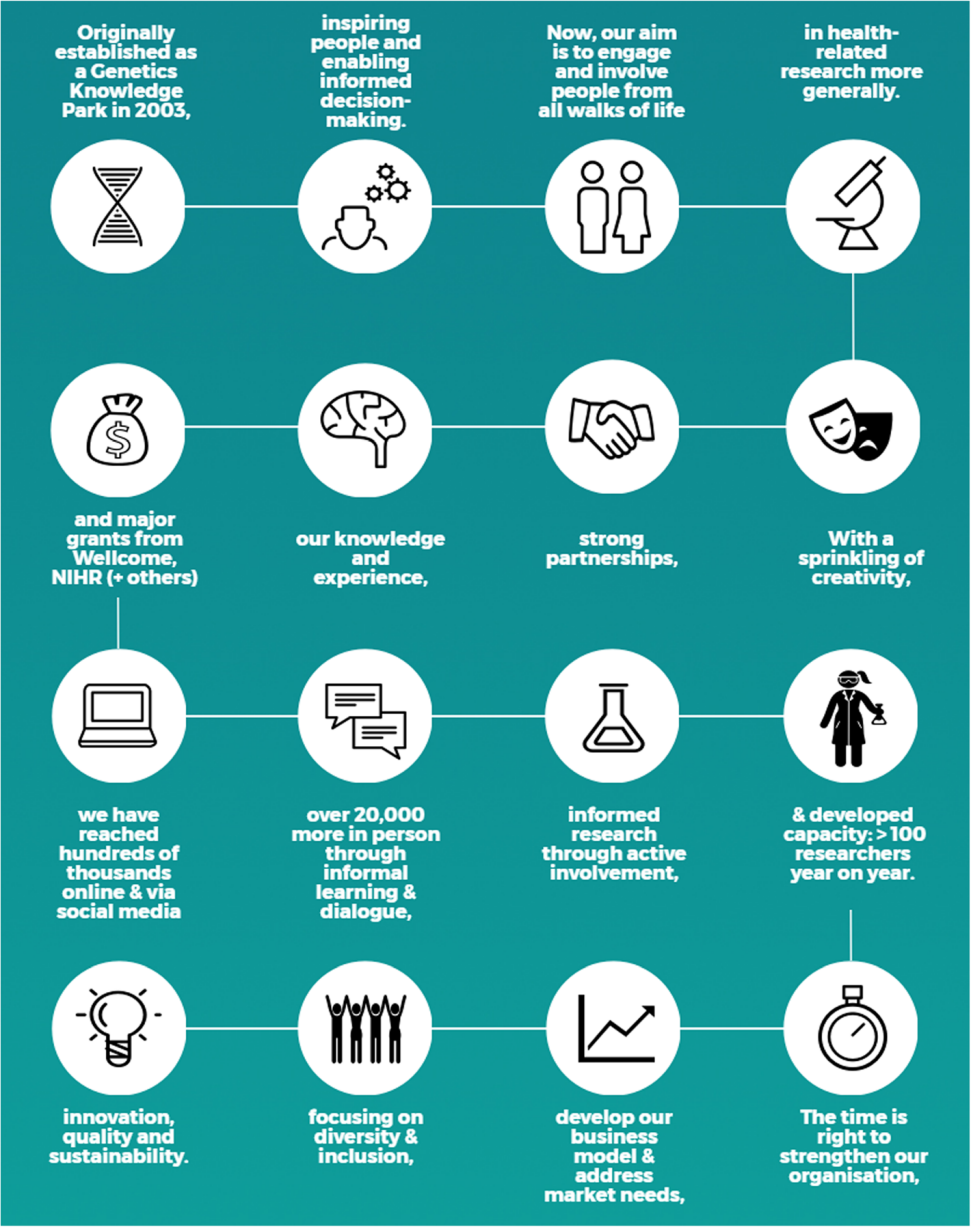
The Public Programmes Story. Originally with a focus on public engagement with genomics, the Public Programmes team has evolved to engage and involve people across health research. The team is embedded within research programmes and leads large scale national initiatives, with a focus on diversity

Since 2003, the Public Programmes team has worked across applied, health service and academic health research environments, supporting and delivering public engagement and involvement, learning and publishing across these sectors (eg. [8–11]) and being recognised through awards from both sectors (eg. [12, 13]). Figure 1 tells our story.

Now a small team of public engagement specialists, with backgrounds in science, community development, public engagement and involvement, policy, ethics, communications, industry, museums and creative practice, we are embedded across translational health research infrastructure and delivery, hosted by Manchester University NHS Trust. The approach and vision of the Public Programmes Team has evolved to: ‘make health research relevant and inclusive for everyone’. Our values, collaboratively established by our team, with input from our partners (including public partners), are defined as: working together; everyone matters; innovating inclusively; driving excellence. We connect and involve people from all walks of life with health-related research, to the benefit of research, researchers, patients and people, and wider research, health and civic sectors. Importantly, the Public Programmes Team adopt a strategic approach to working across engagement and involvement in health research in Greater Manchester. The team receives funding as an integral part of the NIHR Biomedical Research Centre (BRC) and NIHR Clinical Research Facility (CRF), with match funding from the Wellcome Trust and working in partnership with other NIHR infrastructure (eg. NIHR Greater Manchester Patient Safety Translational Research Centre), the University of Manchester and other research groups and organisations, including industry, regionally, nationally and internationally.

Our organisation-wide aims are to:

- Prioritise diversity and inclusion within our contributors, audiences and approaches,
- Experiment with arts-led approaches,
- Innovate across engagement and involvement,
- Deliver best practice evaluation and research informing a continuous cycle of innovation and improvement in our practice,
- Build capacity for engagement and involvement within researchers and public contributors.

### Background – context and opportunities

Our approach builds on a number of contexts and opportunities including: our track record and experience (Fig. 1); already working across public engagement and involvement fields; and a positive shift within PPI in health research to foster more creative and inclusive approaches. It’s clear that multiple methods are emerging to support public involvement (for example, searching for ‘methodology’ within BMC Research Involvement and Engagement alone returned 79 results since the first publication of the journal); a growing confidence in the sector realises that there is no ‘one size fits all’ method (eg. [14]). Another positive shift within public and patient engagement and involvement in health research, recognises the need to understand the impact of engagement and involvement, i.e. The difference engagement and involvement can make to people and to research. Specifically, in Greater Manchester (the second most populous urban area in the UK), the Greater Manchester Health and Social Care budgets became devolved in April 2016, enabling greater collaboration and consolidation across research, healthcare and community landscapes. The region is perceived as ‘testbed’ for working differently and in a more holistic and joined up way across engagement, involvement, research, health and care.

This paper formalises our approach to inclusive research for 2017–2022, by introducing our conceptual ‘cycle’ of engagement and involvement that underpins our practice. We state how we will evaluate our approach, we examine preliminary case studies exemplifying our approach, and report on evidence demonstrating some of its impacts. Finally, we suggest that both our ‘cycle’ and our strategic and collaborative approach present a unique learning opportunity for similar organisations and have the potential to be replicated elsewhere. We are primarily a practice-based organisation and are increasingly aware of the need to publish our approach and work in peer-reviewed publications, even if we are not traditional academics; we have been prompted and encouraged to share our approach through publication by many partners, including NIHR.

## Methods

### Our approach – a ‘cycle’ of engagement and involvement for professional practice

**Fig. 2:**
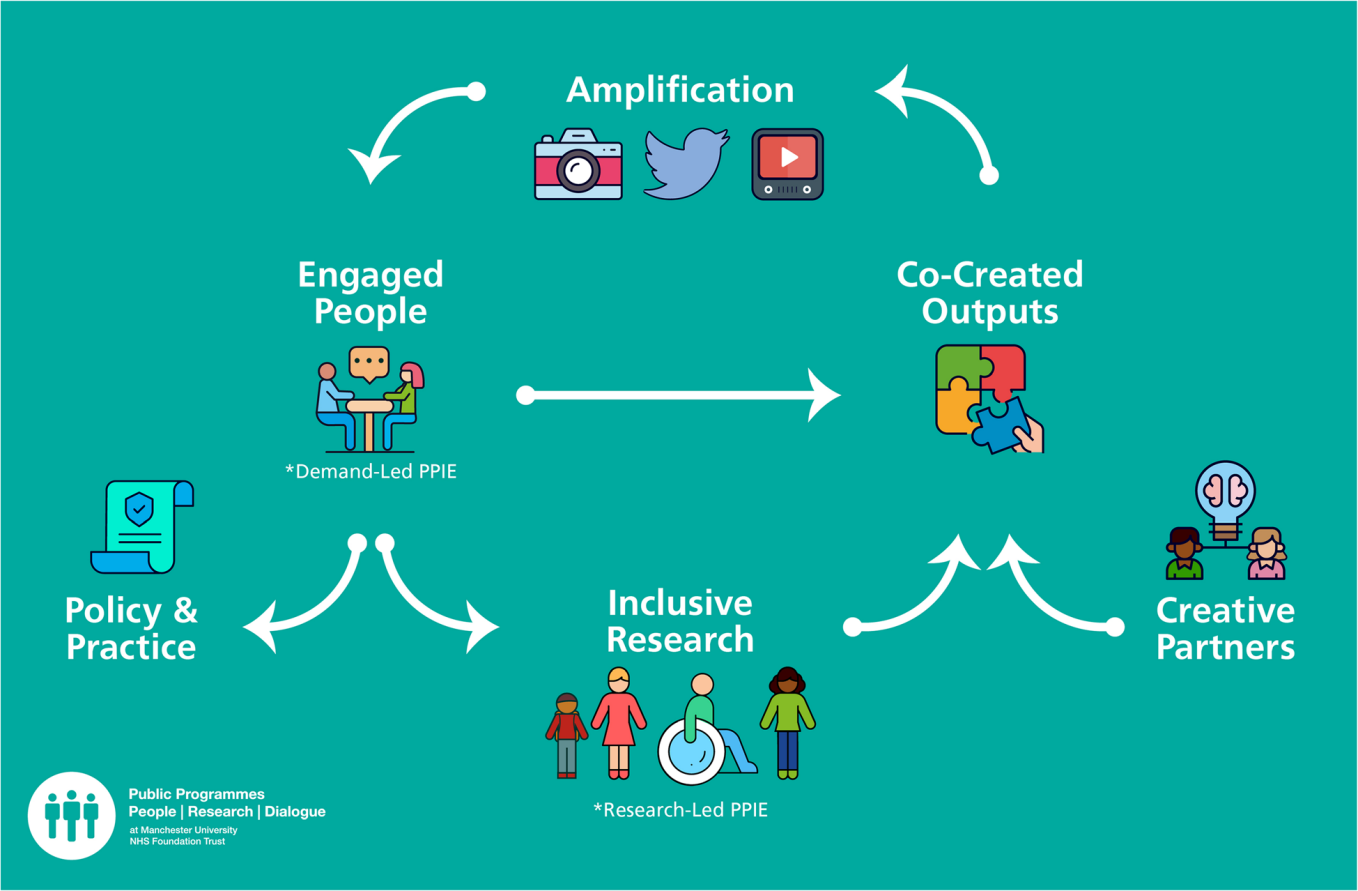
The Public Programmes ‘cycle’ of engagement and involvement. To stimulate an inclusive research community, the cycle revolves around co-creating high-quality engagement outputs that capture curiosity and concerns about research, the amplifying outputs to reach larger audiences. Completing the cycle relies on nurturing and/or establishing progression routes related to health research for all involved, building on engagement

Our approach (Fig. 2) relies on a model stimulating an interactive ‘cycle’ of engagement, involvement and research. Rather than being focused on PE and/or PPI as an outcome in itself, our aim is to stimulate an inclusive research community by being focused on the purposes of engagement and involvement and working appropriately with researchers from different disciplines, people, patients, creative partners and communities from diverse backgrounds and perspectives to achieve clear objectives for working together. Though the methods we might use for connecting people and research will depend on the circumstances, research area, people and patients we’re working with, our cycle of practice revolves around:

- Co-creating high-quality engagement outputs that capture curiosity and concerns about research, by working with creative partners, researchers, research staff, engaged people, and public and patient communities including those who might currently be less well heard in research. In this phase, we focus on arts-led approaches as these recognise and celebrate people’s existing positive social identities — as designers, music-lovers, makers or social activists, for example — and, in our experience, are a key way to invite and welcome people, to engage with research, especially those who might not see science as ‘for them’ [15],
- Amplifying our outputs. This could be through peer-to-peer approaches, broadcast and social media methodologies, and/or partnerships with broadcast, engagement, charity or civil organisations,
- Completing the cycle relies on finding, nurturing, establishing or signposting to progression routes for all those involved.

For engaged audiences, progression routes could be, for example:

- Taking the next step to an active role in health related research (e.g. helping to develop or prioritise research questions with a research team, acting as co-researchers, advising on recruitment through existing mechanisms or through new relationships developed through working together) and/or,
- Contributing to, or leading, further research, engagement and involvement, themselves, and/or,
- Taking part in research, and/or,
- Developing personal agency, confidence, health and research literacy and connections.

For researchers, progression routes could be, for example:

- Increased willingness, knowledge of and capability to work alongside different people and patient groups to influence current or future research and research practice,
- Greater ability to identify, understand, utilise and maximise opportunities for engagement.

For creative practitioners (eg. Musicians, visual artists, radio producers, performance artists), progressions routes could be, for example:

- Stimulating and developing their practice and portfolio to focus on engagement with health and health research,
- Developing connections that are mutually inspiring.

### Our approach: moving from research-led to community-led

Our cycle of practice can be research-led, responding to a demand from the research sector to engage and involve; and/or demand-led (community-led), listening to and responding to people’s and communities’ expectations and thoughts about health-related research. Our demand-led approach complements the ‘human centred’ approach and strategy for engagement recently put forward by the Wellcome Trust [16, 17].

At the outset, our approach will be more research-led. By 2022, we anticipate and hope that our portfolio of work will contain substantially more demand-led activity, thus influencing the research carried out within our context. We are leading this additionally through:

- Working with local communities to inform and co-produce engagement projects and outreach strategies,
- Creating equal power dynamics across PPIE practitioners, researchers and public contributors to understand the needs of audiences when we’re communicating research concepts,
- Involving patients and public contributors in delivering sessions within both engagement and training events,
- Running focus groups for people and patients within engagement events,
- ‘Involved communications’– applying user-testing and co-production in communicating about our organisation, our engagement, our involvement and our research.

We will publish the methodologies and findings of our community-led work in due course.

### Our approach – outcomes and evaluation

We are aware that the outcomes associated with our work are multiple, including:

- Individual empowerment and transformational growth: for all the people involved in our work, including public contributors, creative experts, researchers and others,
- ‘Better’ research - through more inclusive and relevant research practice,
- ‘Better’ engagement and involvement – through more inclusive and relevant practice,
- Increased opportunities for research collaboration and innovation.

Taken together, the above outcomes have the potential to positively contribute to health. Our challenge is to capture impacts and share the learning of a long term body of work and across the range of outcomes listed above. Due to our 5 year approach, we will evaluate each activity, event and project as standard, also routinely collecting monitoring information against our organisation-wide aims across our entire research community (researchers and public contributors).

Our success criteria will focus on:

- Reported changes to research (priorities, design, implementation, analysis, dissemination). This is assessed through: the number of publications evidencing the impact of public involvement; number of public contributors acknowledged in publications, as co-authors, and as co-applicants on research grants; narratives describing the nature and impact of public involvement and engagement on research; and interviews with researchers and public contributors regarding the impact of involvement and engagement,
- Increased diversity of public contributors, recognising the value of intersectional approaches. Through anonymised surveys, developed on advice from the Manchester Urban Collaboration on Health, we have started to capture anonymised demographics of our partners and audiences according to ethnicity, geographical location (postcode), level of education, and other protected characteristics,
- Inclusive practices and environments that are recognised and valued by all. Our community-led strand of work will pioneer and evaluate approaches that will be published in due course,
- Progression routes (in research settings or otherwise) adopted by public contributors and researchers are assessed via survey and through regular qualitative interviews with researchers, creative partners and public contributors, including during project initiation, interim review and final ‘wash up’ meetings,
- Increased knowledge, skills and confidence in PPIE from researchers and contributors, as assessed through regular surveys and interviews (including through an external evaluation consultant) with researchers,
- Increased knowledge, skills and confidence of researchers to work with diverse audiences/contributors, as assessed through regular surveys and interviews with researchers,
- Developing personal agency, confidence, health and research literacy and connections amongst public contributors,
- Extended reach of PPIE and research, as assessed by metrics related to event attendance, communications (eg. Social media reach).

We are working to standardise the evaluation of our PPIE strategy, activities, meetings and feedback processes. A bespoke Customer Relationship Management system is being developed to track and monitor our PPIE, its impacts and progression routes.

## Results

### Results: exemplifying our method

Two case studies (Box 2, Box 3, Additional file 1) presented here exemplify our ‘cycle’ approach to innovating involvement and engagement practice and some of their associated evaluation findings.

**Box 2:** Case study - #DesignforMSK [26] and YourRheum [9, 27]

**What we did**	
In 2016, #DesignforMSK involved people and patients in a creative exploration of the issues faced every day by young patients (16-26 years). Through a patient-led co-design process, the project developed and exhibited design solutions for new, covetable products supporting patients living with musculoskeletal conditions.	
#DesignforMSK and YourRheum demonstrate our ‘cycle’ of engagement and involvement by co-creating, in 3 creative workshops in Manchester, comprising 25 people (young patients, creatives and researchers), 8 product prototypes. Amplification of the outputs came through a physical exhibition at Manchester Art Gallery in December 2016, which also featured artwork created in response to living with invisible disabilities. A digital version of the exhibition was screened at the Museum of Science and Industry in February 2017, broadcast on CMFTV across the hospital campus in early 2017, and digitally engaged audiences through social media reaching over 60,000 people (to February 2017). The project led to the establishment of Your Rheum, a national group for people aged 11 – 24 to advise, input and shape current adolescent and young adult rheumatology research.	
The evaluation of the project reports findings across the success criteria associated with implementation of the ‘cycle’.	
**Young people involved in the project report increased personal confidence and agency within research**	
The emotional and social impact of #DesignforMSK is evident in responses from the young participants, many of whom reported having never met another person their age with similar conditions to their own:	
‘*I think it’s given us the opportunity to not feel so abnormal in this world’ [Young participant 1]*	
‘*After participating in the workshops I now feel I’m not alone and I am glad I can provide support to others’ [Young participant 2]*	
*‘It’s great to be able to speak to people that know what the condition is like and who understand the struggles I have. This has boosted my confidence and I certainly feel less alone now’ [Young participant 1]*	
#DesignforMSK also inspired participants to make a transition from engagement, to pursuing their own involvement in research. Four of the young participants have gone on to become involved with Your Rheum, a national group for people aged 11 – 24 to advise, input and shape current adolescent and young adult rheumatology research. Three of these participants had not been involved in any similar projects prior to #DesignforMSK, and found out about the opportunity directly through being engaged in #DesignforMSK.	
Many conversations during the workshops were about a desire to raise awareness of invisible illnesses and hidden disabilities. Taking part in #DesignforMSK made the participants aware that there are opportunities to become involved in research and affect change. This, and their subsequent involvement in Your Rheum, has motivated the young participants to help others and seek out ways to raise awareness about their condition, research, and having a voice in research:	
*‘I think it’s inspired me to help other people; not just people with our conditions, but anyone with any form of disability’ [Young participant 1]*	
*‘I would like the opportunity for not only Your Rheum members but for all young people with rheumatic conditions to be able to participate in research and to be a lot more informed in how our contribution to research helps’ [Young participant 3]*	
**#DesignforMSK raised awareness of musculoskeletal conditions and research amongst public audiences**	
A major aim of the exhibition was to raise awareness of the fact that arthritis is a condition which affects young people and to raise the profile of this invisible illness and research into it.	
After visiting the exhibition, as assessed through surveys, and word associations, people associated the word ‘elderly’ with arthritis 50% less than before the exhibition; people also felt that arthritis and musculoskeletal conditions were ‘relatable’ after visiting the exhibition. The frequency of use of the words ‘pain’ and ‘debilitating’ decreased after visitors had seen the exhibition, while ‘brave’ and ‘strong’ increased. Survey responses also indicated that the exhibition was successful in its aim of raising awareness of musculoskeletal conditions being something that can affect young people as well as old. It is clear that the exhibition also had a positive impact on people’s understanding of musculoskeletal conditions, making them more relatable to visitors.	
The project had an impressive social medial presence, reaching over 60,000 people.	
**Creative partners reported greater awareness of health conditions on their practice**	
Designers and curators involved in the project all reported an impact on their ways of working and thinking:	
*‘The workshops affected my personal thinking, as I’ve been able to meet people my age who have these conditions, which really touched me and helped me to understand these illnesses and see people in a different way ...’ [Curator]*	
*‘From now on, disabled access to exhibition spaces will be a priority for me’ [Curator]*	
*‘Going forward I will always think about how small changes to my designs could make them more readily available to everyone’ [Designer]*	
*‘I found the workshops gave me much-needed perspective into the experiences of young people with musculoskeletal disorders … I could not help reflecting on how much we take for granted and what it takes for young people with musculoskeletal disorders to engage with society’ [Designer]*	
**Researchers report learning from the project and valued the different perspectives encountered**	
Researchers feedback immediately after the workshops indicated the value of being able to have conversations with people who have musculoskeletal conditions outside of a clinical environment:	
*‘It became clear from the [conversations and activities during the workshop] that their disease affected many aspects of their daily life, but that there was limited information or tools available to help them overcome some of these problems. For us as researchers it was very interesting to listen to all these stories and to go away with possible ideas for future research’ [Musculoskeletal Researcher 1]*	
*‘I came to get an idea of what MSK was, the symptoms and what is currently being done to help patients from a research and treatment point of view. It did give me some answers and also got me thinking about potential research and treatment possibilities.’ [Psychology Researcher]*	
*‘I am currently thinking about doing some research on employment including young adults with Juvenile Idiopathic Arthritis. This workshop emphasised that there is lack of information and [that] people with musculoskeletal diseases encounter various problems during their vocational training and finding their first job.’ [Senior Research Fellow]*	
*‘I also noticed that some of the participants had become friends and they were able to share their ideas and problems which was something I had not thought of as one of the outcomes of the project.’ [Senior Research Fellow]*	
A challenge for the project was researcher recruitment and drop out. Despite this, the young participants enjoyed working with the researchers, although they would have liked to have had more researcher involvement with the project:	
*‘I think it’s fantastic having the chance to work with researchers, and it should be something to aim towards for the future as it gives insight to both participants and researchers about the difficulties we each face’* [*Young Participant*]	
This last point has informed our practice going forward: future projects may benefit from encouraging online discussion between researchers and patients in order to facilitate engagement and to maintain the researcher’s involvement even if they are unable to attend face-to-face meetings; and clear communication about a project’s aims and potential outcomes.	
The Research Advisory Group YourRheum continues to thrive.

**Box 3:** Case study - Summary of #BreathtakingLungs [21]

**What we did**	
#BreathtakingLungs [Additional file 1 & 20] took a place-based approach to exemplifying and testing our ‘cycle’ of engagement and involvement. Wythenshawe in Greater Manchester has the largest clinical respiratory department in the UK. The surrounding areas also have significantly higher rates of respiratory conditions than nationally and a poor health profile, according to the Manchester City Council Neighbourhood Profile.	
Over a 6 month period in 2018, 16 people with lived experience of respiratory conditions and living in Wythenshawe, researchers, an artist, community workers and the Public Programmes Team co-created a range of engagement outputs including:	
• Singing workshops: 15 people with breathing conditions met every week to sing, carry out practical breathing exercises, and discuss research with researchers who also took part in the singing,	
• Harmonica sessions: accessible sessions run in libraries and community locations, including content on respiratory conditions and research,	
• A youth project working with 15 young people using graffiti and focused on air pollution (Wythenshawe is close to Manchester airport), creating a powerful mural,	
• A fully functioning lung model, particularly popular with children and families,	
• Breathing Blue: an immersive artistic response, featuring local people’s stories and voices, raising awareness of lung conditions and research. The wearable sculptures engaged audiences in out-patient centres, town centre and community location.	
The ‘core’ partnership group engaged 127 people in 29 activities about breathing, breathlessness and research. The amplification phase of the ‘cycle’ came through further Wythenshawe community events engaging approximately 550 people. Further amplification came through social media, reaching over 99,940 Twitter accounts in the recorded period, media appearances on local and regional radio and TV, and touring Breathing Blue at the Manchester Science Festival, and other engagement and cultural events. Several of the project participants have taken steps to active participation and involvement in research, closing the ‘cycle’.	
The evaluation of the project reports findings across the success criteria associated with implementation of the ‘cycle’.	
**Participants report reduced social isolation and positive health outcomes**	
Participants in the co-creation groups reported how the project and its activities had ‘got them out’. They reported that prior to Breathtaking Lungs they did not always leave their house regularly. People who took part in the wider project activities talked of the benefits of being part of a group and improvements in their capacity to manage their illness, both through interactions with health and research professionals, and through learning from other participants.	
*‘We’ve done art, we have done mouth organs, we sing, in fact for me the last six weeks have been a new start of life for me.’ [Participant 1]*	
*‘It’s helped me breathe better and I love it.’ [Participant 2]*	
They also reported improvements in self-esteem, from a sense of achievement of developing a new skill (eg. Singing or harmonica playing) and improvements in physical strength. These findings are especially pertinent when considering the health profile of the area.	
**Communities and families report greater awareness of research and research involvement**	
Many of the people reached by the project reported positive perceptions of research feeling that it was needed, that the benefits to future population were ‘enormous’ and that therefore it was important to consider volunteering as a research participant. People reported that the project had increased their awareness of lung health and lung research. They also reported sharing this knowledge and awareness with family and friends, and that it may have helped them to explain their conditions to loved ones. The range of age groups involved in Breathtaking Lungs meant that it was easy for older and young members of the same family to be involved in the project.	
*‘And bringing my granddaughter in as well, that made it doubly good.’ [Participant 3]*	
The group also talked very positively about the role of the Clean Air Day event ran at Wythenshawe Forum on raising awareness of lung health locally. They felt that it played an important role in increasing people’s curiosity about and awareness of lung health and that the event had been beneficial locally in term of raising awareness of lung health (the availability of lung health testing was considered to be particularly important).	
The creative and informal format of the engagement sessions and activities was especially valued by the people taking part:	
*‘The best thing about playing today, it was informative, it helped with the breathing but it was good fun and I really, really enjoyed it.’ [Participant 3]*	
*‘I loved the sense of friendliness and I loved the information and access I had.’ [Participant 5]*	
People reached by the Breathtaking Lungs project have since gone on to become involved in respiratory patient panels and providing written feedback on research proposals and information documents, further events run by the Public Programmes Team, the VoiceUp Youth Research Advisory Group run by the Public Programmes Team and participating in research studies. Two people are now also currently involved in helping develop the pilot ‘Breathe Better’ drop-in support sessions with the NHS Community Respiratory Teams.	
**Researchers valued creative conversations**	
People reached by Breathtaking Lungs valued contact with researchers and would have welcomed more opportunities to talk with researchers:	
*‘We actually even met Professor Vestbo who’s the Professor at Wythenshawe Hospital doing research and he actually put himself on the hot seat and had us fire these questions and he was absolutely amazing.’ [Participant 2]*	
Researchers valued the ‘open and honest conversations’ brokered by the project, as did the participants, who particularly appreciated the care taken by the Public Programmes Team to create formats that ‘levelled the playing field’ between researchers and public and patient audiences, having relaxed and informative conversations, different to the ones they might have in clinic:	
*‘I liked that everyone wasn’t afraid to express their opinion.’ [Participant 4]*	
The project developed a number of engagement outputs which continue to be used by the researchers involved in Breathtaking Lungs and the Public Programmes Team.	

### Results: key preliminary achievements

**Fig. 3:**
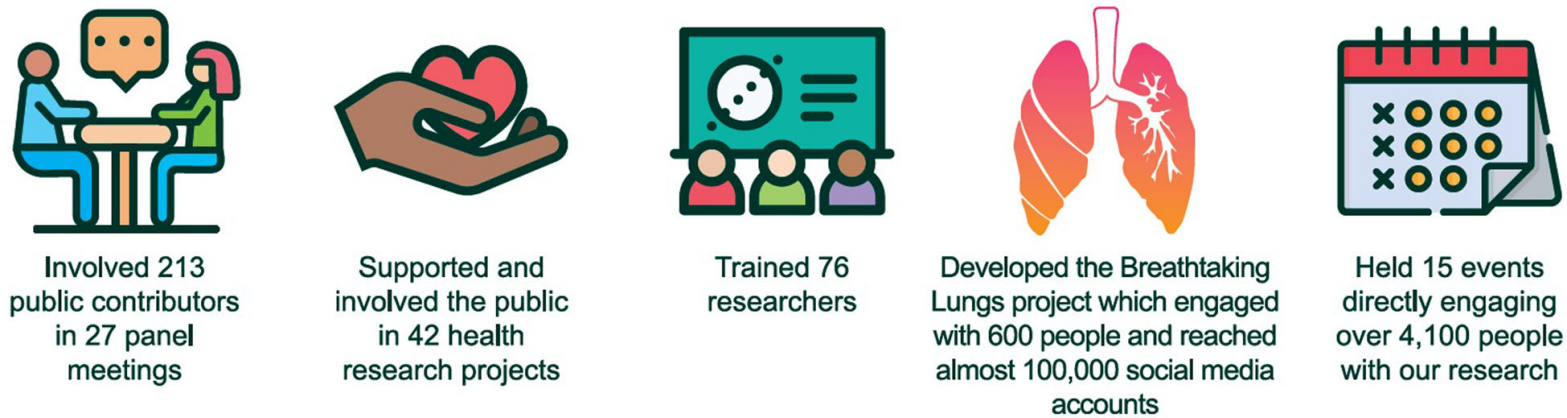
Summary of PPIE achievements in Year 1. Visual summary of public and patient involvement and engagement (PPIE) outputs achieved by the Public Programmes team from April 2017 to March 2018

We are just at the beginning of testing our approach; our early (Year 1; Fig. 3) notable achievements, and continuing challenges, include:

*A joint PPIE strategy across translational research infrastructure*: working intensively with research, public and patient partners, we have elaborated a joint PPIE strategy [18] across the Public Programmes team, Manchester BRC, Manchester CRF and the Research & Innovation Division of the Manchester University NHS Foundation Trust (and in partnership with the University of Manchester). Over 100 staff, researchers and contributors contributed. Other research partners with whom we collaborate also sign up to our strategic aims.

*Working with a large and varied group of public contributors*: We have recruited and embedded public contributors for monthly Executive Group, quarterly Governance Board, and additional strategic meetings. In Year 1, we have worked directly with over 250 public contributors in a variety of ways (eg. As part of governance, or on specific projects).

*Flourishing our research-led involvement and co-production of research.* 213 public contributors were involved in 27 bespoke face-to-face patient ‘panel’ involvement meetings serving specialties within BRC and CRF themes. Alongside meetings, a broader online approach for either light-touch digital, or response-mode involvement work has been developed. 42 research projects have involved public contributors, including, for example, the first UK trial of Proton Beam Therapy [19]; experience-based co-design work with the Manchester CRF [20] and the development of a Deaf Experts by Experience Group to advise on hearing health research.

*A focus on young people*: Voice Up – a young people’s research advisory panel with approximately 70 members from diverse demographics, and representing every Borough of Greater Manchester – has been set up and included as part of the GenerationR network of Young People’s Advisory Groups.

*Engaging numerous people:* Our research-led engagement has held over 15 events and directly reached over 4000 people directly (and several hundred thousand more online and via social media) to raise awareness of our research, including opportunities to get involved or participate. Significant projects include: Breathtaking Lungs [21] (Box 3, Additional file 1); The Future in your Hands - a touring photography exhibition developed with musculoskeletal patients [22]; and the 100 Voices project [23], as well as multiple open days, engagement events in collaboration with creative, community and research partners across Greater Manchester.

*Developing capacity for researchers*: we have delivered training for 76 research staff, advised researchers on 16 grant applications and implemented a baseline survey of researcher training needs. By bringing NIHR-funded and Higher Education PPIE leads from across the North of England for a one-day workshop, and establishing a local forum for Cancer PPIE practitioners, we have stimulated networks and communities of practice to share learning.

*Celebrating our research community* – including our public contributors, researchers and creative partners. Our research community is our greatest asset and a highlight of Year 1 was a ‘tea party’ held for 72 people to say ‘thank you’ to our research community, in particular, our public contributors.

*Beginning to understand the progression routes taken by everyone we work with* – although this is at an early stage. We continue to document progression routes of all involved.

*Pioneering demand–led and inclusion PPIE work*, by developed relationships with over 40 community voluntary sector organisations across Greater Manchester. 5 projects have been delivered with young people from Wai-Yin Chinese Society, Ananna (a Bangladeshi Women’s group), Safe Ambition, Reform Radio [24], and #ThisVibrantThing festival. A successful collaboration has been established and funded by the Wellcome Trust, engaging and involving Jewish communities in pregnancy and placental research. A two-day Community Innovation event in July 2018, working with Greater Manchester Black and Minority Ethnic network. All will be reported on in due course.

## Discussion

This paper sets out to: firstly, introduce a unique approach to working innovatively and inclusively across the fields of public engagement and patient involvement through a new ‘cycle’ model of professional practice. Secondly, it reports preliminary results that exemplify and demonstrate the utility, applicability and impact of the model. Thirdly, it explains the wider context of the strategic and collaborative approach undertaken by the Public Programmes team. Finally, we suggest that both the ‘cycle’ and the Public Programmes Team’s strategic and collaborative approach have the potential to be replicated and applied elsewhere. We believe that the collateral knowledge generated through our approach has the potential to benefit research, researchers, people, communities and health.

### Testing the ‘cycle’ of engagement and involvement

The case studies present projects and their evaluation which positively address the success criteria associated with the ‘cycle’ model (see Methods) and therefore suggest that the model could present an effective way of bridging PE and PPI to foster more inclusive research. The case studies (Box 2, Box 3, Additional file 1), and other projects described in the Results section, reported:

- Changes to research, for example through the establishment of a national research advisory group (YourRheum) including people who had taken part in #DesignforMSK; changes to research protocols through involvement (eg. [19]),
- Increased diversity of public contributors: most of the public contributors who progressed from ‘engaged people’ (Fig. 2) to people actively involved in research, had never connected with health research before and represented communities and constituencies underrepresented in public involvement,
- The value of creative approaches as ‘entry points’ to engagement and involvement. For example, at least two of the young adults in #DesignforMSK (who then progressed to members of YourRheum) describe that they would not have taken part in the project had it not had a creative element,
- Increased knowledge, skills and confidence in PPIE from researchers and contributors, as witnessed by researcher comments,
- Extended reach of PPIE and research, as demonstrated by the impressive numbers of people directly and indirectly engaged (eg. through social and mainstream media) by #BreathtakingLungs, #DesignforMSK and other projects in Year 1,
- People and patients developing agency, confidence, health, research literacy and, in some cases, positive health outcomes. For example, participants in #BreathtakingLungs reported reduced social isolation and being able to breathe more easily as a result of the engagement activities. Reduced social isolation was a strong feature reported by the young participants in #DesignforMSK. This finding echoes a wider arts and health agenda linking cultural activities and public health (eg. [25]).

### Embedding PPIE and inclusive research

At a more programmatic level, our evaluation (so far) and experience points to a growing culture change, embedding PPIE within research, and according it a high-level status within translational research infrastructure. The challenge remains to continuously ensure that different voices are supported to be heard, within reporting, executive and governance structures that are sometimes less flexible than specific projects. Another challenge remains in acknowledging, planning and building in the time and effort – in particular from researchers, with many demands on their time already – required for effective PPIE. Whilst our team facilitates this ‘on the ground’ and through our strategic, joined up and practical approach, researcher involvement is crucial and often compromised by other demands.

Shifting the emphasis of PPIE from research-led towards community-led will take time. A challenge for our Team will be in maintaining relationships with communities and organisations, understanding that trust requires continued engagement especially in the context of austerity and pressures on community organisations. The balance between developing trust and programmes of co-produced work, as well as delivering on research-led commitments, remains acute.

### A strategic and collaborative approach

Working through strategic collaboration has enabled the Public Programmes team to operate as a financially self-sufficient, semi-independent organisation, underpinned and hosted by the Manchester University NHS Foundation Trust. Furthermore, the approach has successfully leveraged funding for joint PPIE initiatives across the range of research organisations partnered with the Public Programmes Team. Through this way of working and business model (which has the potential to build up financial reserves and streamline processes), the Public Programmes Team achieves a joined up approach to plan and deliver patient and public involvement and engagement at size and scale. This allows economies of scale and continuous improvement. For example, working across several research organisations with a unifying strategy, allows a strong presence, a ‘critical mass’ of multiple research groups at public and community events, that can be coordinated by one team. Working across multiple organisations further allows for effective shared learning and capacity development.

Going forwards, we are committed to reporting on and sharing our progress, through NIHR and funding and reporting requirements, through publications such as these and our less academic communications (e.g. YouTube channel) and events. We have been encouraged to disseminate our approach through publication by many including NIHR, even though we are not traditional academics. This is the start. We hope you enjoy it.

## Conclusion

This paper outlines the approaches taken by the Public Programmes team at Manchester University NHS Foundation Trust – a team of public involvement and engagement specialists, embedded within translational health research infrastructure and delivery in Greater Manchester, UK – to innovate inclusive research, engagement and involvement, through strategic collaboration and practice. Our evolving practice and the beginning of its evaluation lead us to believe that our way of working is effective in: addressing our vision of making health research relevant and inclusive for everyone; embedding and joining up public involvement in a busy and fertile health research ecosystem; addressing our strategic priorities of diversity and inclusion, working with arts-led approaches, delivering gold standard evaluation and building capacity for engagement and involvement within researchers and public contributors.

## Additional file

Additional file 1: Case study - Summary of #BreathtakingLungs [21]. (PDF 782 kb)

## Data Availability

Not Applicable

## Abbreviations

MBRC: Manchester Biomedical Research Centre
MCRF: Manchester Clinical Research Facility
PPIE: Public and Patient Involvement and Engagement
