# Supplementary material for: Innovating public engagement and patient involvement through strategic collaboration and practice
Source: Res Involv Engagem. 2019 Oct 21;5:30. doi: 10.1186/s40900-019-0160-4 (PMC6802177; doi:10.1186/s40900-019-0160-4)
Supplement: Supplementary file 1 — Case study - Summary of #BreathtakingLungs [21]. (PDF 782 kb) [file 40900_2019_160_MOESM1_ESM.pdf]

#BREATHTAKING

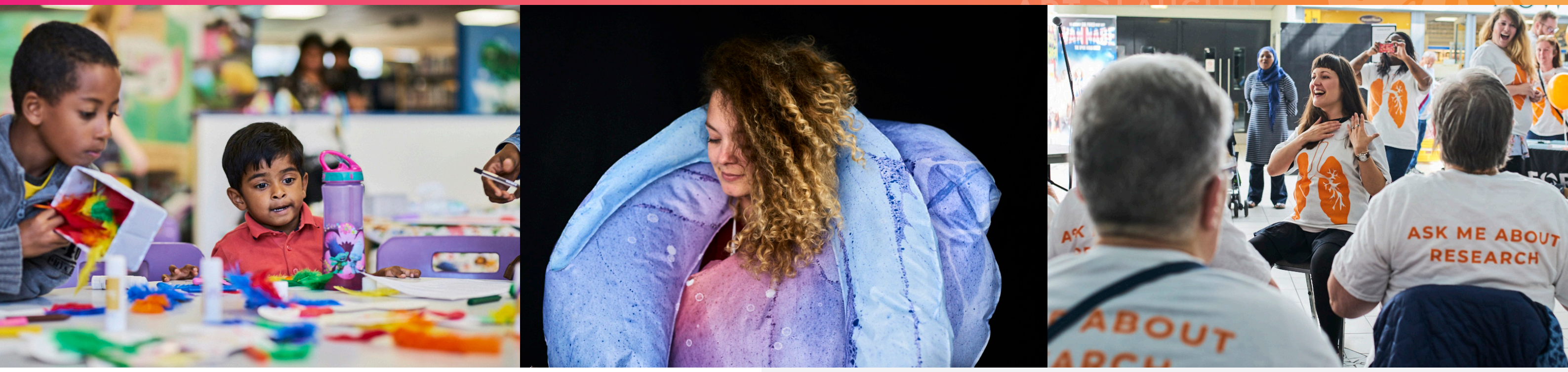

Clean Air Day: Family craft workshops, *Breathing Blue* wearable artwork, singing group performance   Photos: Rob Battersby

The context

Wythenshawe has the largest clinical respiratory department in the UK. It also has significantly higher than average rates of respiratory conditions, yet the voices of this local community seem to be largely unheard. Our aim was to change this. We used arts-based and community-led approaches to reach and engage with people who might never have connected with health research. We also brought patients and researchers together.

Our approach

From January to June 2018, the Public Programmes Team worked with researchers, patients and the public, artists and community partners in Wythenshawe to raise awareness of lung health, breathing conditions and related research through a range of creative activities. Sixteen people living with a range of respiratory conditions, took part in focus groups to help develop the project ideas and the communications plan.

> **"We've done art, we've done mouth organs, we sing, in fact for me the last six weeks have been a new start of life for me."**
>
> **Marian**, Breathtaking Lungs participant

Highlights

- Engaged **127** people in **29** singing, art, music and discussion-based activities about breathing, breathlessness and research
- Engaged approximately **550** people at community events at The University of Manchester and Wythenshawe Forum
- Reached a large audience through social media with **196,949 impressions** achieved on Twitter reaching a total of **99,940 accounts**
- Gained local and regional media attention with appearances on **Wythenshawe FM**, **BBC North West Tonight** and **BBC Radio Manchester**
- Since the project, participants have been advising researchers as part of patient panels, are helping to establish a new social event for patients with COPD and have been involved in a range of other research activities.

#BreathtakingLungs
breathtakinglungs.co.uk
publicprogrammes@mft.nhs.uk
0161 276 6614
PublicProgrammesTeam
@researchdialog

Funded by NIHR
